# Reducing healthcare access inequities in West Java through forecasting and location-allocation models

**DOI:** 10.1186/s12913-026-14889-1

**Published:** 2026-06-10

**Authors:** Mursyid Hasan Basri, Rhys Price, Paul Harper, Lukman Hilfi, Daniel Gartner, Syaribah Brice

**Affiliations:** 1 Faculty of Economics and Business, Universitas Bangka Belitung, Pangkalpinang, Indonesia; 2 School of Mathematics, Cardiff University, Cardiff, UK; 3 Department of Public Health, Faculty of Medicine, Universitas Padjadjaran, Bandung, Indonesia; 4 Aalen University, Aalen, Germany; 5 Cardiff and Vale University Health Board, Cardiff, UK; 6 Department of Mathematics, Universitas Negeri Malang, Malang, Indonesia

**Keywords:** Facility location, Forecasting, Optimisation, Community care, Underserved populations

## Abstract

**Background:**

Ensuring equitable access to healthcare services is paramount for the enhancement of societal welfare and the optimisation of public health outcomes. Within the context of West Java Indonesia, accelerated population growth coupled with geographic disparities has resulted in heightened pressure on the existing healthcare infrastructure, thereby rendering numerous communities inadequately served. Although CHCs are already widespread in Indonesia, their distribution remains uneven, especially in growing urban areas. Consequently, there is growing recognition of the need to strategically expand community healthcare centres (CHCs) to improve access across underserved areas. These CHCs are instrumental in providing vital services, encompassing preventive care, maternal and child health, immunisation, dental services, and outpatient care.

**Methods:**

This study presents an integrated approach combining population forecasting, spatial demand estimation, and optimisation modelling to support long-term planning of CHCs in Kota Bandung. Kota Bandung was selected as the study area due to its high population density, significant spatial disparities in healthcare access, and strategic importance in regional health planning. Using an ARIMA model, the researchers forecasted population growth through 2045. The year aligns with Indonesia’s national long-term vision, *Visi Indonesia 2045*. They then estimated daily CHC demand using spatial grid and population density data, applying a standard utilisation rate. Three mixed-integer linear programming (MILP) models were developed to optimize facility locations by maximizing coverage, minimizing travel time, or balancing both. This method allows planners to explore various scenarios based on local needs and constraints.

**Results:**

The forecasting model reveals a consistent escalation in the demand for primary healthcare services within the Bandung region, with distinct growth hotspots identified in presently underserved locales. Optimisation scenarios produced by the MILP models indicate that the strategic positioning of new CHCs can substantially augment service coverage while simultaneously mitigating disparities in healthcare access. Scenario-based simulations were conducted to explore how different planning objectives—such as maximizing coverage or minimizing travel time—impact CHC placement outcomes. The results suggest that the most equitable arrangement of CHCs would enhance accessibility for marginalized populations by more closely aligning resources with geospatial demand.

**Conclusion:**

This integrated modelling framework presents a pragmatic paradigm for long-term health infrastructure planning in contexts characterized by resource limitations. By synthesizing demand forecasting with location-optimisation methodologies, regional health authorities are empowered to make data-informed decisions to enhance access and equity in primary healthcare services. These findings bolster broader initiatives aimed at achieving Universal Health Coverage in Indonesia.

**Supplementary Information:**

The online version contains supplementary material available at 10.1186/s12913-026-14889-1.

## Background

Indonesia, with a growing population of more than 280 million, is the world’s fourth most populous country [1]. The World Health Organization has observed variability in coverage and associated barriers to accessing healthcare services for many Indonesian populations due to factors such as economic instability and geographic location [2]. Consequently, a particular concern for those planning for and delivering healthcare services across the country is ensuring that a sufficient system is put in place to provide all citizens with the necessary medical support and easy access to service.

With a population of approximately 50 million, the province of West Java has the largest population of all 38 Indonesian provinces and a high population density, second only to the country’s capital Jakarta [3]. The continuous growth of West Java’s population is expected to sharply increase by 2045 due to factors such as urbanization, migration from smaller provinces and natural birth rates [4]. As the population rises, it is critical to plan for the increased strain that will be placed on the already struggling healthcare system in West Java [5] to improve both access to healthcare and health equity within the region.

The Indonesian healthcare system runs on a hierarchical multitiered system, utilizing a large network of both public healthcare facilities and privately funded providers [6]. In 2014, the Health Social Security Agency (*Badan Penyelenggara Jaminan Sosial Kesehatan*,* BPJS Kesehatan)* implemented a National Health Insurance scheme (*Jaminan Kesehatan Nasional*, JKN). The JKN program aimed to achieve universal health coverage for all Indonesian citizens, resulting in a substantial increase in healthcare utilisation across the country. The introduction of the JKN programme has been widely regarded as a success by the Indonesian Ministry of Health [7] and the World Bank [8] in that there has been a significant increase in the utilisation of healthcare services in Indonesia [9].

However, rapid urbanization and increasing population growth are currently approximately 0.84% annually, leading to a projected population of over 300 million by 2045 [10]. Hence, Indonesian healthcare services are being placed under significant strain. This has led to overcrowded facilities, long waiting lists and access to services, especially impacting underserved populations such as those who are severely geographically disadvantaged within this large county, which comprises more than 17,000 separate islands [11]. Strategically expanding healthcare infrastructure through the placement of new CHCs is therefore essential to meet rising demand and reduce spatial inequities in access. Future research may further explore how existing facilities can be upgraded or reallocated to complement this expansion.

Working closely with the West Java Provincial Health Office, our work utilizes operational research methods to help the health authority with an expressed initial need to model primary care community facilities in the region of Kota Bandung, serving a population of just under 3 million. Primary care services have been chosen because they act as the foundation of the healthcare system, providing a first point of contact for most Indonesians [12]. Primary care facilities provide treatment for common illnesses, administer vaccines and immunisations when required and aim to prevent the spread of disease. Although CHCs are already widespread across Indonesia, their distribution remains uneven, particularly in rapidly growing urban and peri-urban areas. The primary care provision is mainly facilitated by *Puskesmas* or otherwise known as CHCs (Community Healthcare Centres). These CHCs provide crucial care, such as preventative, maternal and child services, immunisations, dental care and outpatient services [13]. In addition, they also act as a referral point for any case that may require more specialized care to be sent to secondary or tertiary facilities. While this study focuses on Indonesia, the challenges of urban population growth, geographic health disparities, and infrastructure constraints are common to many low- and middle-income countries. Community healthcare centres (CHCs) have been widely adopted in various global contexts as effective frontline providers, especially in decentralized health systems aiming to improve access and achieve Universal Health Coverage. Several other countries have also implemented similar models. For instance, Brazil’s Family Health Strategy has demonstrated significant success in improving population health through community-based primary care teams [14]. Likewise, community health centres in Canada have been shown to effectively serve underserved populations through integrated, team-based approaches [15].

The purpose of our work was to develop a modelling framework that can support the provincial health office in the strategic planning of primary care facilities. This includes exploring both population forecasting and optimisation of facility locations to maximize coverage and minimize travel distance—thereby directly addressing access gaps among underserved populations. Moreover, the modelling approach can be easily parameterized and applied to all regions and cities within West Java. Given similar service structures and data availability, the framework could also be adapted for use in other provinces across Indonesia.

To achieve these goals, we implement a multistage approach:

1. Population forecasts: development of a model to provide population forecasts until 2045.
2. Demand estimations: development of a method to convert population forecasts into geospatial demand estimates for primary care services.
3. Location-allocation optimisation: development and application of models to help evaluate and compare the geographical provision of primary care services that (a) maximize healthcare coverage, (b) minimize patient travel distances, and (c) balance coverage and accessibility via a weighted hybrid model.

Challenges faced by working with our partners in West Java and thus overcoming these challenges led to aspects of novelty in our research, including limited demand data. This required us to develop alternative methods to estimate geospatial demand by combining our population forecasts with available population density maps. Our mixed-integer linear programming location-allocation models incorporate a novel formulation to ensure that all demand is assigned. This approach allows demand nodes to be allocated to more than one facility. Each facility can serve multiple demand nodes. Such flexibility ensures that every facility is utilised to its maximum efficiency. Another feature of our research was the development of all our decision support tools via accessible open software where possible, thus avoiding the need for costly licenses that are out of the reach of our collaborators in Indonesia. For example, we make use of OpenStreetMap data and the OSMnx library in Python to estimate travel times and distances combined with a coded Dijkstra’s algorithm to avoid the need for expensive travel time software and with the optimisation and mapping models created via a bespoke Excel spreadsheet via OpenSolver.

### Literature review

This section reviews the existing literature on facility location and healthcare access planning, with particular focus on methods that integrate optimisation and forecasting techniques. The following studies provide the theoretical and methodological foundations for the modelling framework developed in this paper.

Facility location problems and their derivations have long been studied in the literature, with a wide range of applications. Facility location decisions are a critical element in strategic planning for many industries and governmental bodies and directly impact operational and logistical decisions. In particular [16], provided a comprehensive review of literature that explicitly addresses the strategic nature of facility location problems, especially those involving stochastic or dynamic characteristics. Additionally [17], offered an overview of fifty years of methodological developments and applications in location analysis.

A helpful overview of published work for both capacitated and uncapacitated facility location problems is provided by [18]. The uncapacitated facility location problem (UFLP), which is one in which planning for locations of facilities does not need to explicitly consider constraints on the number to locate, includes examples dating back many years, such as [19], who compared and contrasted a developed heuristic for warehouse location with published attempts to solve the problem through simulation or a variation of linear programming (LP). Other examples of methods include those based on branch and bound algorithms [20], tabu search [21], variable neighborhood search metaheuristics [22] and discrete event simulation [23].

The capacitated facility location problem (CFLP) requires the use of optimisation and heuristic methods to determine the best locations for facilities while considering capacity constraints and minimizing total costs. Various approaches have been employed over the years, such as enhancing Bender’s decomposition performance in mixed integer applications [24], a hybridized method of genetic algorithms and subgradient techniques [25] and a combined two-phase possibilistic LP approach with a fuzzy analytical hierarchical process (AHP) [26].

As with other sectors, healthcare facility location problems have been considered in the literature for a range of problems, such as locating vaccination Centres during the COVID-19 pandemic [27], preventative health Centres [28], obstetric care units in Korea [29], and organ transplant Centres [30]. A recent comprehensive review of OR methods applied to a range of healthcare problems, including facility locations, was provided by [31].

Within the broader facility location literature, location-allocation problems specifically focus on simultaneously locating facilities and allocating demand points to facilities, with a range of applications, especially in urban planning environments [32]. Despite the large number of studies, surprisingly few papers specifically consider location allocation for future demand, and instead, most consider the current situation with known demand of the system they are looking to improve [33]. Given that the aim of our work is to ensure that the correct facilities are created to ensure that a sustainable healthcare system is in place for the rapid expansion of demand that might be expected until 2045, this would suggest a need to integrate forecasting techniques with location-allocation approaches.

There are various methods for examining location-allocation problems. Famously, models such as the *p*-median model and maximal coverage location problem (MCLP) have been used as foundations for the creation of specific models. The MCLP, first introduced by [34], aims to maximize the coverage of demand with as few facilities as possible, where demand is considered ‘covered’ if it falls within a user-defined distance. The MCLP has been commonly used when looking at problems such as ambulance response times and police arrivals, where the primary concern is the worst-case scenario, with multiple heuristic approaches being implemented to solve these problems, such as [35, 36]. While this method has been widely adapted [37], introduced a new approach to the problem through the addition of facility capacities, leading to the capacitated maximal covering location problem (CMCLP), which links the uncapacitated and capacitated models with general assignment problems.

 [38] extended CMCLP work to incorporate workload limits on facilities. Whereas the authors consider only one facility per demand node, we propose that demand nodes be assigned to more than one facility as needed.

When looking at minimizing distance or travel time, the *p*-median model is a commonly used base for mixed-integer linear programming. Originally introduced by [39], the model aims to minimize the sum of distances between demand nodes given the total number of facilities that are needed. The *p*-median model assumes that all facilities have equal capacity and unlimited service capability, which makes it inappropriate for our task; however, authors such as [40] created a variation of the capacitated *p*-median model that enables facility limitations, which is more suited to our needs. Other heuristic approaches, including genetic algorithms, have been used, including [41, 42].

With a lack of location-allocation literature specifically focused on future demand, only a few examples exist, including [43], who combined demographic forecasting for healthcare facilities ensuring coverage and accessibility for anticipated population changes, and [44], who incorporated population growth estimates to determine how the population size and distribution would impact service coverage and response times.

This study contributes to that limited body of work by explicitly combining long-term population forecasting with flexible location-allocation modelling, offering a scalable and data-driven framework to address spatial inequities in access to primary healthcare. While promising for strategic planning, the study is subject to certain limitations—particularly the lack of real-time utilisation data and the simplifying assumption of equal service capacity across proposed CHCs.

## Methods

As discussed in the introduction, the aim of our work was to develop a strategic modelling framework that can assist the provincial health office in the long-term planning of primary care facilities. A particular feature of our approach is the use of scenario-based simulation up to 2045, which involves forecasting demand and applying location-allocation models to maximize coverage while minimizing travel distance. This framework enables planners to evaluate multiple future planning options and directly address disparities in underserved populations.

This section describes the development of a forecasting demand tool that comprises population forecasts that feed into daily geospatial CHC demand estimates (via the creation of a ‘demand grid’) for Kota Bandung. These findings are then used to parameterize three mixed integer linear programs (MILPs) that are constructed to permit the evaluation and comparison of the geographical provisions of CHCs in West Java.

### Population forecasts

One of the most widely used models for forecasting time series data such as population growth is the ARIMA (Autoregressive Integrated Moving Average) model, known for its relative simplicity and accuracy when applied to historical trends [45, 46]. We selected ARIMA over alternative methods such as exponential smoothing or ARIMAX because the available population data lacked robust exogenous predictors (e.g. migration, birth rates) at the required spatial resolution. As noted by Box et al. [46], ARIMA is particularly effective when only univariate historical data is available, while Benevenuto et al. [45] show its applicability in regional health service forecasting.

**Fig. 1:**
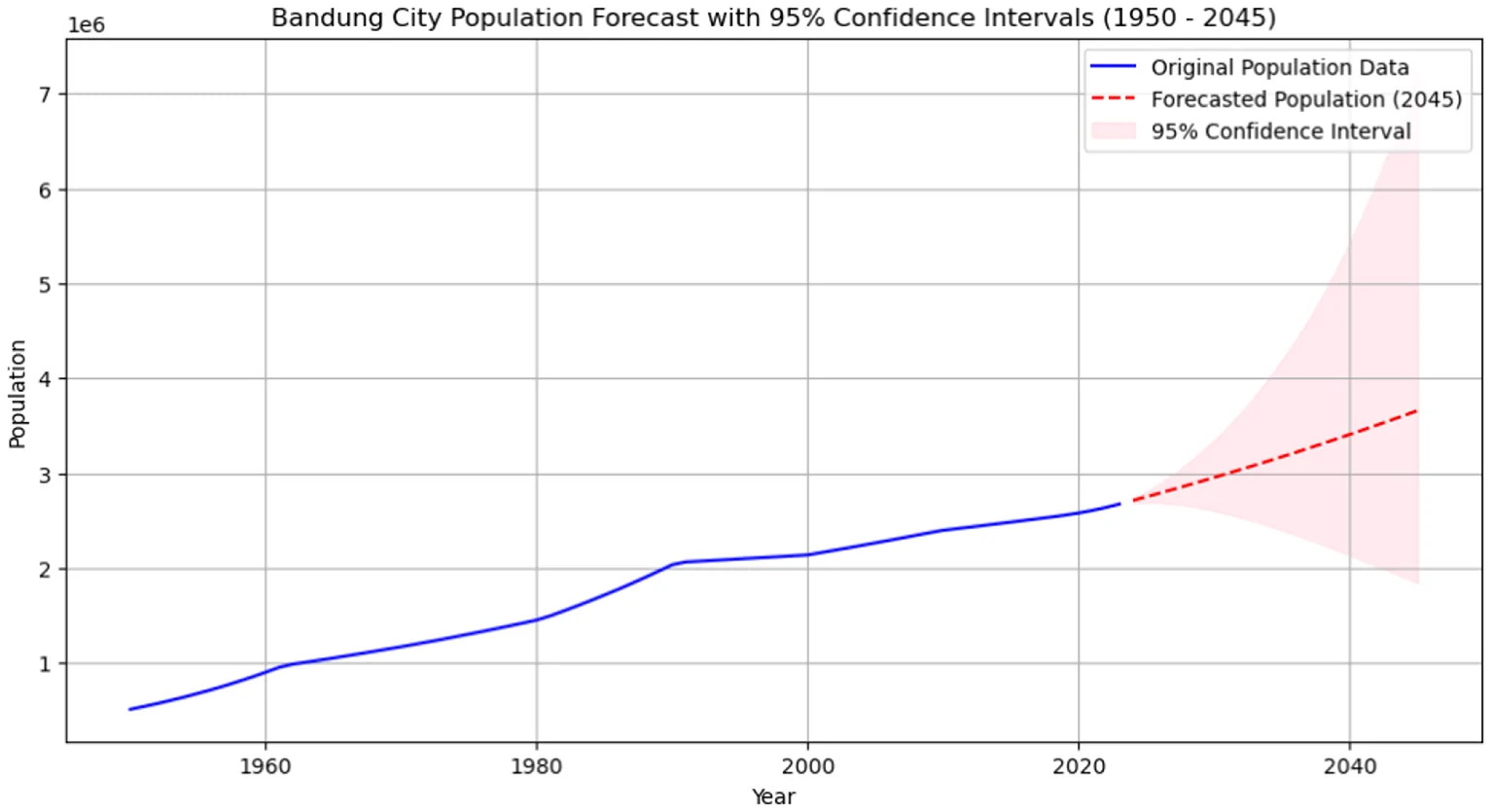
Kota Bandung population data and forecasts (in millions) from 1950–2045

The ARIMA model was implemented using population data from the World Population Prospects dataset, which offered the most consistent historical record for the Bandung area. Forecasting was conducted through 2045 to align with Indonesia’s national development vision. The training dataset spanned from 1950 to 2015, with test data from 2016 to 2023. The model was evaluated using Mean Absolute Error (MAE), Root Mean Square Error (RMSE), and information criteria (AIC, BIC, HQIC). The selected model, ARIMA(0,2,1), was found to produce the best fit based on these indicators. The model selection process, including the stationary tests and correlation analysis (ACF and PACF plots), is detailed in Appendix 1. Forecast results, including 95% confidence intervals, are presented in Fig. 1, while model comparisons and selection metrics are reported in Table 1.

**Table 1:** ARIMA model parameterization and fit

Model	AIC	BIC	HQIC
ARIMA(1,2,1)	-588.234	-581.404	-585.515
ARIMA(1,2,2)	-586.075	-576.968	-582.449
**ARIMA(0**,**2**,**1)**	**-590**,**247**	**-585.693**	**-588.434**
ARIMA(0,2,0)	-577.286	-575.009	-576.379

Although the confidence interval (1.8–7.2 million by 2045) appears wide, this reflects the compounded uncertainty over a 22-year horizon and remains within acceptable bounds for long-term strategic planning. Moreover, while other models such as ARIMAX or machine learning–based forecasting may bypass strict stationarity requirements, they were excluded due to lack of consistent covariates and explainability constraints. The non-stationarity issue was addressed through second-order differencing, after which the Augmented Dickey-Fuller (ADF) test confirmed stationarity (i.e., a sufficiently negative statistic, not non-stationarity, as previously misstated).

### Demand estimation

Building on the population forecasts, we now estimate future daily demand for CHCs, allocate this demand geospatially and then use this demand to find the numbers and best locations of CHCs to cover the demand.

Our next challenge, however, was a lack of data on current CHC demand at levels lower than districts (which are rather high-level regions). This led to the concept of a *demand grid* as a collection of *demand nodes* equally spaced out over the region under investigation. For Kota Bandung, the demand grid created was a 25 × 25 grid covering approximately 1,100 km^2^. This resolution was selected as a balance between spatial granularity and computational feasibility, ensuring that each 2 × 2 km² node could reflect localized demand without overwhelming the optimisation model. Regional demand node polygons (each with a size of 4 km^2^) and their exact locations (longitude and latitude coordinates) were obtained from https://geojson.io and were used to help decide which polygons were contained within the Kota Bandung boundary; furthermore, these polygons were used as locations for generating demand in the optimisation work. The granularity of each demand node polygon (as a 2 × 2 km^2^ area) was set and was deemed sufficient for forecasting local demand and planning services in the absence of reliable data.

**Fig. 2:**
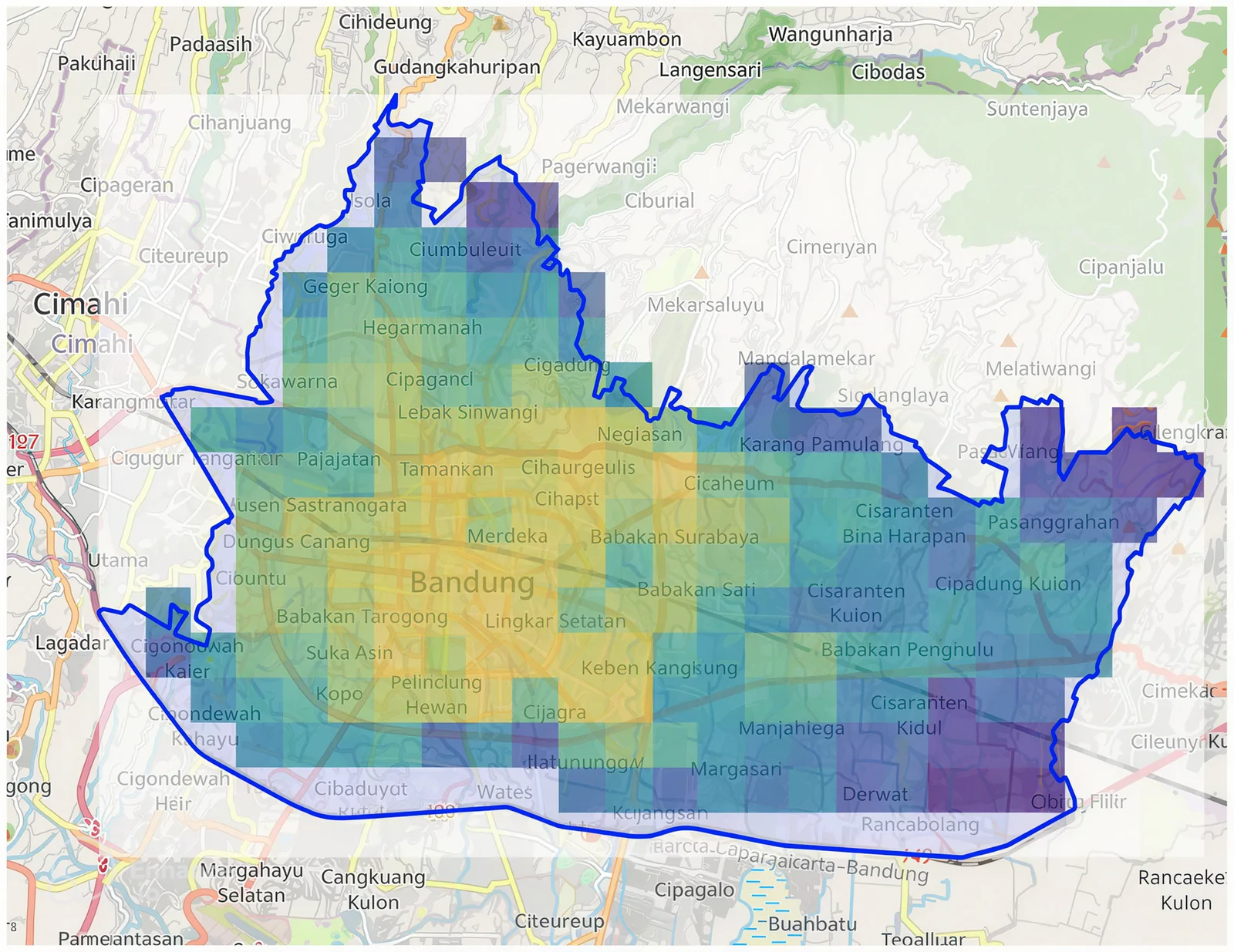
Kota Bandung population density heatmap

With the demand nodes confirmed, to find the daily demand forecasts for each node, we first need to estimate the population that is represented by each node. To do this, reliable population density data covering Indonesia from [47] were spliced over the region and transformed into a population density heatmap, as shown in Fig. 2. The lighter the shade of the node is, the higher the population density is, as shown in Fig. 2. As expected, the density is greater toward the Bandung city center region and lower closer to the Kota Bandung region boundary, which is more rural in nature.

Now, we have the population density, and assuming that the population density does not significantly change until 2045, we distribute the population forecasted in the ARIMA forecasts over the demand nodes according to the population density in the area in which they reside. While this assumption simplifies the modelling process, we acknowledge that it is a strong one. In reality, urban development policies, infrastructure investments, and socio-economic shifts may cause certain areas to experience faster densification or depopulation. However, in the absence of detailed, spatially granular projections or zoning-based urban expansion plans, a static density assumption was necessary for generating baseline demand estimations. It allows the model to serve as a foundation for strategic planning, with flexibility to incorporate updated population distribution inputs as they become available. We convert the population into expected daily demand, albeit we acknowledge somewhat crudely, but in the absence of data less than at the district level, we were only able to use known published demand data from [48], which reported a CHC utilisation rate (to the population size) of 1.671% per day. Using this approach, we approximated the demand that would be required by each demand point by multiplying the estimated population at each demand node by the published utilisation rate. This led to an overall estimate in 2045 of 60,294 daily visits to primary care facilities (assuming they operate, as planned, 6 days a week). This estimate was considered sufficiently reasonable by the research team based on its consistency with observed utilisation patterns reported in [48], and was thus used as a basis for the optimisation work. Naturally, a recommendation of our work was also to ensure more routine collection of regional CHC demand to help with future modelling, but nevertheless, the modelling framework developed in this paper allows for changes to parameter values and various possible scenarios to be explored, as discussed later in this paper. Although the derived dataset contains no missing values, it remains a synthetic approximation due to the absence of individual-level CHC visit records, gender- or age-specific demand, or verified facility usage rates.

### Location-allocation models

This section describes the development of three mixed-integer linear programming (MILP) models. The rationale for the three models was to experiment with different objective functions and present the West Java Provincial Health Office with a range of scenarios to aid their decision-making depending on what they felt was the most important consideration.

As described in the introduction, most of the primary care in Kota Bandung is provided by *Puskesmas* or otherwise known as CHCs (community healthcare Centres). There exists in the region, however, another primary care facility called a *Clinic*, which tends to be smaller than CHCs and privately funded, although it can accommodate publicly funded patients if required and where capacity is available (typically up to 100 patients per day). When considering the future capacity of primary care services, the Provincial Health Office expressed the desire to consider both CHCs and Clinics within the service provision, each with their own specified daily capacity; hence, this is reflected in our location-allocation modelling.

The first developed model aims to maximize the coverage of facilities to reach the largest demand possible, the second model aims to minimize the travel time for patients to reach their assigned primary care facility, and the third model is a weighted model that is designed to consider both coverage and travel times based on user-defined preferences/weights.

In constructing the models, we require multiple sets, indices, parameters and decision variables, as presented below:

Sets, indices and parameters

**Table:** 

I	The set of all demand nodes in the region
*J*	The set of all facilities and potential facility sites in the region
*i*	Index of the demand nodes
*j*	Index of the facility and potential facility locations
*a*_*i*_	The demand volume from demand node *i*
*t*_*ij*_	The distance between demand node *i* and facility *j*
*c*_*ij*_	Coverage value: a binary parameter that indicates whether facility *j* can cover demand node *i* and is set to 1 if facility *j* does cover demand node *i* or 0 otherwise.
*p*	The maximum number of facilities that can be opened.
*K*_*1*_	The capacity of facility type 1 (CHCs)
*K*_*2*_	The capacity of facility type 2 (Clinics)
α	A weighting parameter that determines the relative importance of minimizing the distance and maximizing the coverage.

Decision variables

**Table:** 

x_ij_	A continuous decision variable between 0 and 1 that represents the proportion of demand from node i that is assigned to facility location j
*y*_*j*_	A binary decision variable set to 1 if facility is open at node *j* or 0 otherwise.
*z*_*j*_	A binary decision variable set to 1 if facility type 1 is to be selected or 0 otherwise.
*d*_*ij*_	A continuous variable that represents the amount of demand from node *i* that is served by facility *j*.

#### Model 1: Maximizing patient demand coverage

This MILP is adapted from [38] by allowing demand nodes to assign their demand to multiple facilities and for each facility to be assigned to multiple demand nodes. The objective function is to maximize the demand assigned to facilities based on the allocated facility number. Objective function (1) maximizes that the demand from the nodes of a region is covered across all facilities.

1 $$\rm \:Maximise\:\sum\limits_{i \in \:I} {\sum\limits_{j \in \:J} {{c_{ij}}{d_{ij}}} } $$

The following constraints consider the different types of primary care service facilities (CHCs and Clinics), the limits on the number of each type of facility, the necessity for each demand node to be served, and the capacity of each facility. Constraint (2) ensures that the number of facilities opened cannot exceed the specified maximum.

2 $$\rm\:\sum\limits_{j \in \:J} {{y_j} \leq \:p} $$

Constraint (3) ensures that for every demand node *i*, all demand at that node is satisfied by a facility.

3 $$\rm\:\sum\limits_{j \in \:J} {{d_{ij}} = {a_i}\:\:\:\forall \:i \in \:I} $$

Constraint (4) links the demand served by facility *j* to the facility type assigned to facility *j*. This ensures that each facility *j* can only be assigned demand to a maximum of its total capacity.

4 $$\rm\:\sum\limits_{i \in \:I} {{d_{ij}} \leqslant \:{K_1}\left( {1 - {z_j}} \right) + {K_2}{z_j}\:\:\:\:\:\:\forall \:j \in \:J} $$

Constraint (5) ensures that no demand is assigned to facility *j* unless that facility is operated.

5 $$\:{x}_{ij}\le\:{y}_{j}\:\:\:\:\:\:\:\:\:\:\:\:\:\:\:\:\:\forall\:i\in\:I,\:\forall\:j\in\:J$$

Constraints (6–9) set the decision variables as binary, and constraint (8) sets the decision variable *d*_*ij*_ to nonnegative, ensuring that a negative demand can never be assigned to a facility.

6 $$\:{\:y}_{i},{z}_{j}\in\:\left\{\mathrm{0,1}\right\},\:\forall\:i\in\:I,\:\forall\:j\in\:J$$

7 $$\:{x}_{ij}\in\:\left[\mathrm{0,1}\right],\:\forall\:i\in\:I,\:\forall\:j\in\:J$$

8 $$\:{d}_{ij}\ge\:0,\:\forall\:i\in\:I,\:j\in\:J$$

9 $$\:{x}_{ij}\in\:\left[\mathrm{0,1}\right]\in\:I,\:\forall\:j\in\:J$$

#### Model 2: Minimizing distance traveled by patients

This MILP model is an adaptation of the commonly known *p*-median model [39]. It aims to minimize the distance traveled for the patients from each demand node, assigning demand to nearby facilities with available capacity. As with the previous model, this MILP has been modified to allow demand nodes to assign their demand to multiple facilities and facilities to take demand from multiple demand nodes.

The objective function for this model is to minimize the distance that each patient must travel to reach their assigned facility:

10 $$\rm\:Min\sum\limits_{i \in \:I} {\sum\limits_{j \in \:J} {{t_{ij}}{x_{ij}}} } $$

subject to constraints (2)-(9).

Constructing the required distance matrix between demand node *i* and facility *j* presented a significant challenge, as commercial travel time software was prohibitively expensive for the provincial health office. Instead, to overcome the prohibitive cost of commercial travel time software, we combined freely available OpenStreetMap data with the open-source OSMnx Python library and a customized implementation of Dijkstra’s algorithm to compute realistic road network distances. This approach is innovative in the context of health service planning in low-resource settings, where proprietary GIS tools are often inaccessible.

**Fig. 3:**
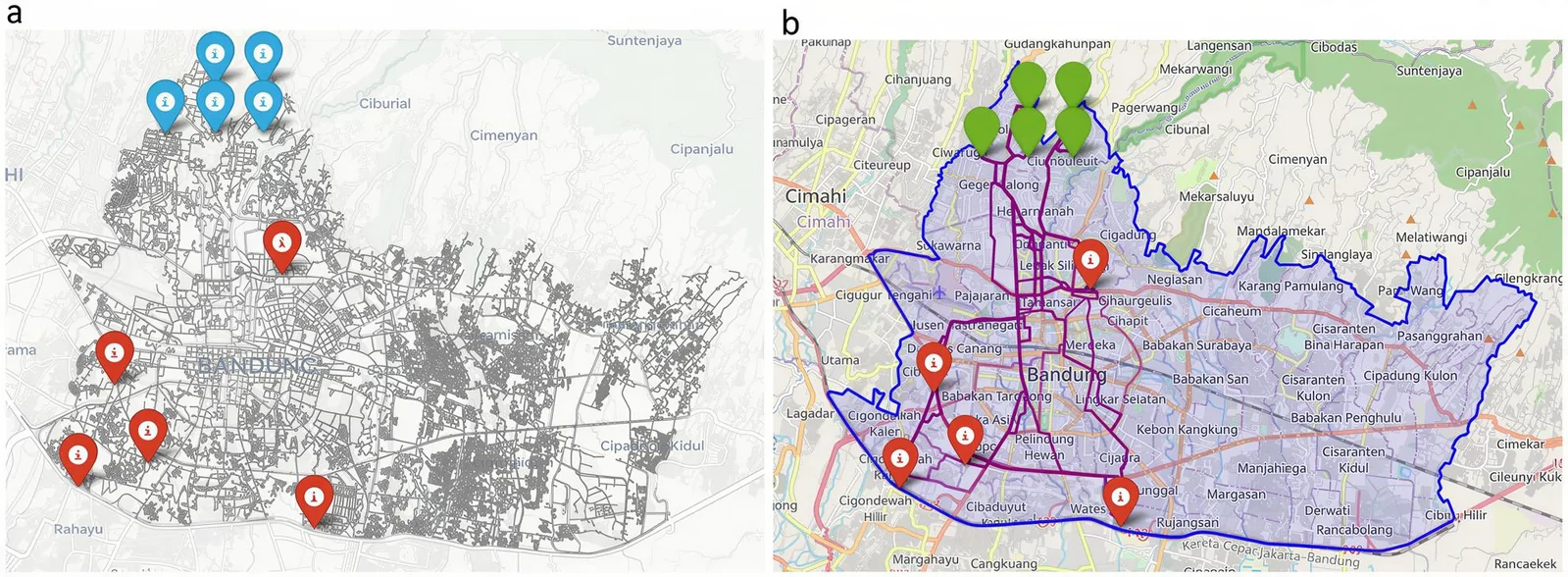
**A**: Kota Bandung road network. **B**: Example of Dijkstra’s algorithm

A graph of the road network in the Kota Bandung region was created, as shown in Fig. 3. The roads were restricted to ‘drive’, meaning that only the routes that were accessible by car were added to the graph. Every demand and facility node were mapped to the closest road on the graph via the *nearest_nodes* function from *osmnx.distance*. Once the road network was set up, to find the distance between every node in set *I* (demand nodes) and set *J* (potential facility sites), we used Dijkstra’s algorithm, which computes the shortest possible path between each node.

Figure 3 includes, simply by way of illustration, five demand nodes (from the demand grid) in the northern region and 5 potential facility locations (to the south). For such a scenario, our developed code first maps the road network (shown in Fig. 3a) with road distance segments taken from OpenStreetMap data and then uses Dijkstra’s algorithm to compute the shortest route for each demand - facility pair (shown in Fig. 3b), hence outputting a 5 × 5 distance matrix (in km) from each demand node to each facility.

#### Model 3: Weighted sum model to minimize distance and maximize coverage

Model 3 is designed to be a combination of models 1 and 2, thus facilitating the exploration of decision-makers for both distance traveled and coverage. The importance assigned to each (minimize distance and maximize coverage) is defined by the decision maker via a weight $\:\alpha\:\in\:\left[\mathrm{0,1}\right]$.

The combined objective function integrates both the distance minimization and coverage maximization components via a weighted sum approach:

11 $$\rm\:Min\:\:\alpha \:(\sum\limits_{i \in \:I} {\sum\limits_{j \in \:J} {{t_{ij}}{x_{ij}}} } ) - \left( {1 - \alpha \:} \right)\left( {\sum\limits_{i \in \:I} {\sum\limits_{j \in \:J} {{c_{ij}}{d_{ij}}} } } \right)$$

subject to (2)-(9).

All three optimisation models were run on a specially designed Excel spreadsheet with a user-friendly interface, and the COIN-OR CBC solver engine was deployed.

### Model assumptions, parametrization and optimisation results

Working closely in collaboration with the West Java Provincial Health Office, this section describes the parametrization and use of the optimisation models, interfaced with the reported findings from the forecasting and corresponding demand estimation.

All three models have been parameterized based on the following features and assumptions, which of course could easily be modified for future work should these changes. The models assume that all newly planned CHCs have uniform capacity and that existing CHCs are fixed in location and operate at average utilisation rates. While CHC throughput may vary depending on staffing and service mix, this abstraction was necessary to focus the model on spatial equity and access coverage.

Additionally, the models do not account for stochastic elements (e.g., fluctuating daily demand, service interruptions), and do not constrain the closure or transformation of existing facilities. These simplifications were made to ensure the model could support rapid scenario testing and planning dialogue. Future work could explore stochastic programming approaches and incorporate dynamic constraints tied to budgeting or facility transition plans.

- All CHC facilities are designed with similar resources that have the same capacity of 480 patients per working day and open 6 days a week.
- Clinic facilities can accommodate at most 100 patients per working day, opening 6 days per week. This estimate assumes each facility operates for 8 h per day, with two doctors seeing patients for approximately 10 min per consultation—resulting in a capacity close to 100 patients per day per facility.
- There are already 188 primary care facilities whose locations are assumed to be correct. These facilities can be modified if necessary to have the same resource levels and meet the upper limit on daily patient demand as stated above; otherwise, they can be closed. New facilities can be built where a need is identified.
- A total of 279 locations were identified as candidate facility sites, which included 188 existing facilities with up to 89 additional locations, as needed. Their locations have been determined on the basis of the availability of land and development potential, with an additional working assumption that each of these new facilities could be a CHC given their greater capacity than private Clinic.
- An initial service standard with a coverage distance of 50 km is desired, i.e., no patient should travel more than 50 km to their allocated facility.
- In addition to the 50 km maximum distance, a median patient travel time of 30 min would also be desirable, if possible. In the absence of costly travel time/distance software, to translate distances into travel time estimates, the average travel time in the Kota Bandung region is taken to be 11.8 km/h on the basis of the best available reliable data source [49].
- To ensure a robust and sustainable future provision of primary care and given that the development of these facilities and associated investment by the health office is once in a generation event (strategic planning resources), the demand for service should be based on the predicted population size and daily demand estimates for the year 2045, i.e., a 20-year ahead plan.
- The population forecast for the Kota Bandung region, until 2045, is given by the ARIMA(0,2,1) model, with a population density (Fig. 2) assumed not to significantly change over the next 20 years. For example, if major housing development takes place in the future, then the models could be easily rerun to capture the modified population density map and hence changes in geographical demand.
- The use of the demand grid approach and the above assumption of population density results in a total of 91 demand nodes with corresponding estimates of daily demand from each node.

We present results based first on the predicted ‘likely’ demand from the ARIMA model and second for a range of varying demand scenarios, including low (-20%), moderate (+ 10%), and high (+ 30%) demand forecasts to reflect uncertainty in population growth and service utilisation rates.

The optimisation models were implemented in Python using the PuLP library with the CBC solver. Computations were performed on a standard laptop with an Intel Core i7 processor and 16GB of RAM. Across the three scenarios, typical solver runtimes were under 5 min per run. The optimality gap reported by the solver was 0.0%, indicating that globally optimal solutions were found. While robustness against parameter changes (e.g., demand shifts) is not explicitly tested in this paper, the model structure allows for flexible re-runs with adjusted inputs.

## Results

### Model outcomes across scenarios

The population forecast derived from the ARIMA(0,2,1) model estimates that Kota Bandung will reach approximately 3,658,887 people by 2045, generating an expected 60,294 daily visits to community healthcare centres (CHCs). Demand was distributed across 91 demand nodes and 279 candidate facility sites, of which 188 are currently operational.

#### Model 1: Maximizing patient demand coverage

**Fig. 4:**
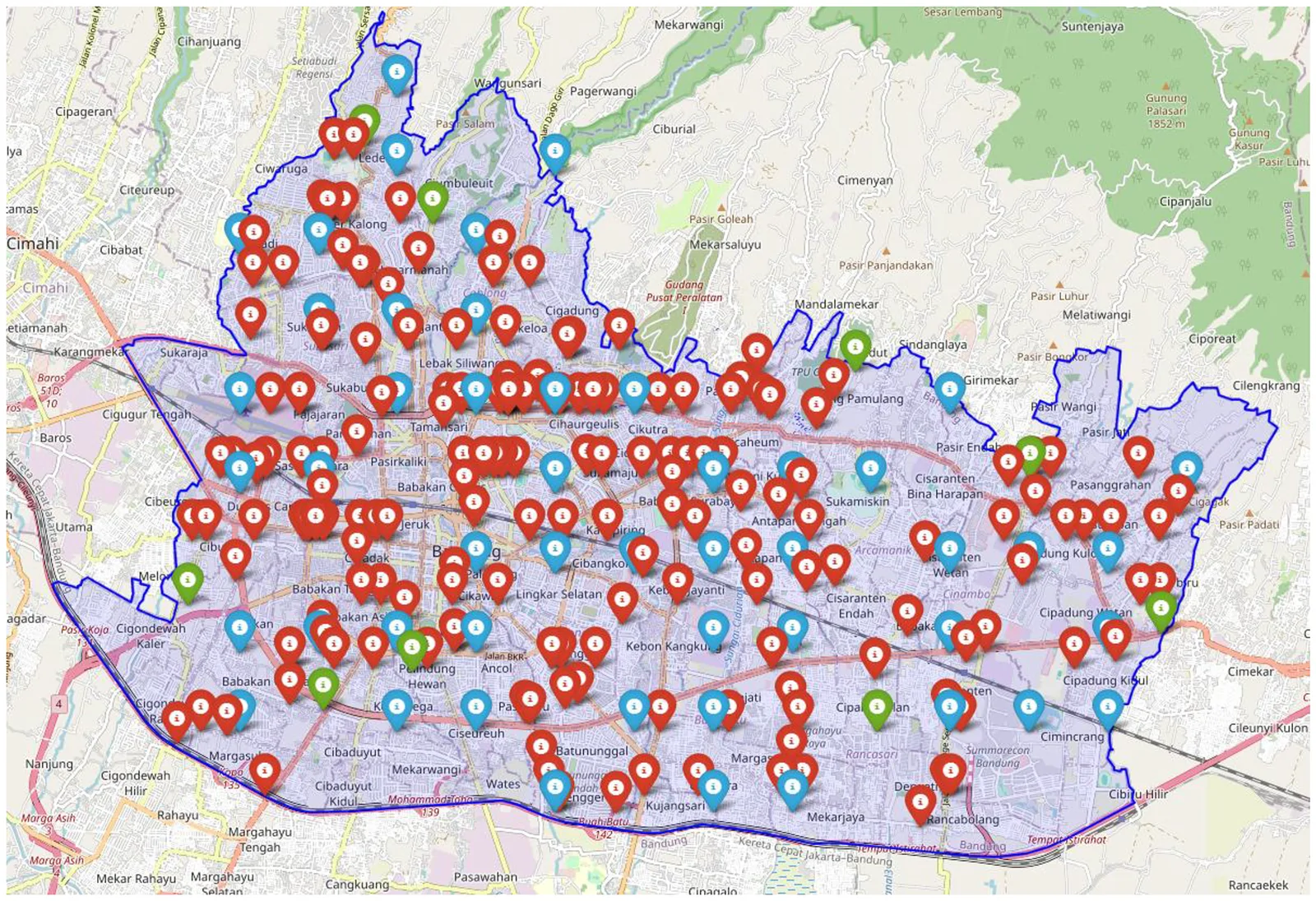
Likely demand scenario for maximizing coverage (Model 1)

The maximum coverage model (M1) prioritized serving the largest possible share of the population within a 50 km service radius. The optimal solution selected 223 facilities, requiring the opening of 44 new CHCs while recommending the closure of 9 existing Clinics. This configuration ensured near-complete spatial coverage, although average travel times remained relatively higher due to dispersed siting across peripheral areas. Figure 4 illustrates the geographical distribution of the chosen facility locations, with red nodes representing retained facilities, blue nodes representing new CHCs, and green nodes indicating Clinics that would no longer be used.

#### Model 2: Minimizing patient travel distance

**Fig. 5:**
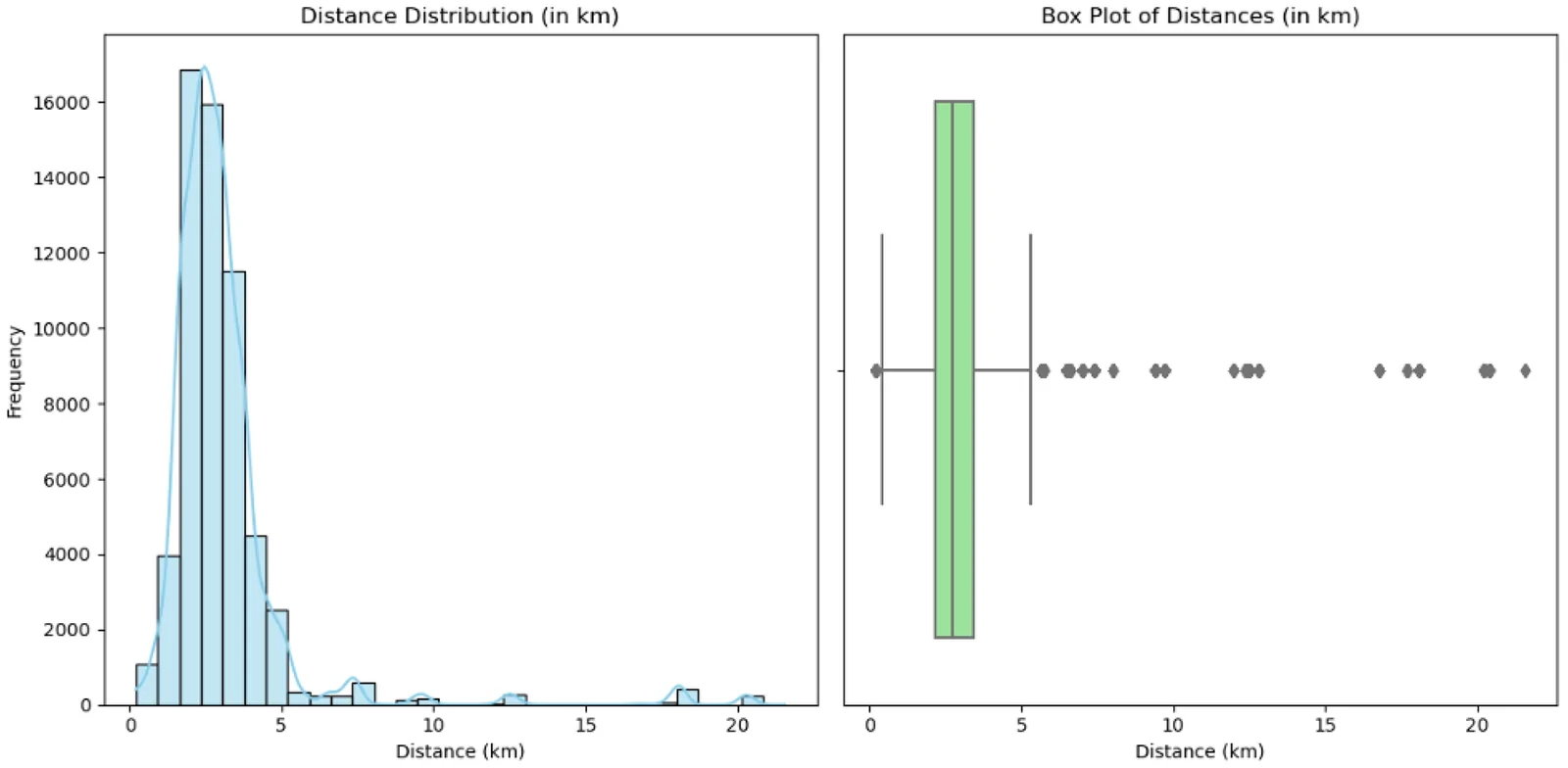
Likely demand scenario for minimizing travel distance (Model 2)

The minimum distance model (M2) sought to reduce travel burdens for patients. Constraining the number of open facilities to the 223 identified in Model 1, the configuration concentrated service provision in high-density central areas. The results yielded a median travel distance of 2.71 km (13.8 min), with a maximum travel distance of 21.6 km (110 min), all within the desired service thresholds. However, this came at the expense of poorer coverage in peripheral, sparsely populated zones. Figure 5 presents the distance distribution and boxplot under this scenario.

#### Model 3: Balanced model (coverage–distance trade-off)

The balanced model (M3) provided a compromise between coverage and travel time by adjusting the weighting parameter (α). With *p* = 223 facilities, this model produced an equilibrium solution that maintained broad coverage while controlling travel burdens. Table 2 summarizes the results across three weightings of α, showing the trade-offs between coverage maximization and distance minimization.

**Table 2:** Likely demand for weighted preferences (Model 3) with three different values of α

Weighting	Optimal facilities	Median distance traveled (km)	Median time traveled (mins)	Maximum distance traveled (km)	Maximum time traveled (mins)	Interquartile range (km)
α = 0.25	223	4.67	23.7	21.59	109.8	2.88
α = 0.50	223	2.71	13.8	21.56	109.6	1.23
α = 0.75	223	2.71	13.8	21.56	109.6	1.29

These baseline results provide the foundation for understanding how different planning objectives influence service provision. While each model outcome is presented individually in the Results, their comparative implications and robustness are explored further in the following Discussion.

## Discussion

This study demonstrates how integrating long-term population forecasting with location–allocation modelling can inform strategic planning for community healthcare centres (CHCs) in rapidly urbanizing regions. The results reveal clear trade-offs between equity and efficiency: maximizing coverage ensures near-universal access but requires more facilities and longer travel times, while minimizing distance improves convenience for central populations but leaves peripheral areas underserved. A balanced approach provides a practical compromise, highlighting the importance of context-sensitive decision-making. To further interpret these findings, we first compare the baseline model outcomes side by side before examining extended facility options and varying demand scenarios.

### Comparative analysis of baseline models

To synthesize the baseline findings, we compared the outcomes of the three optimisation models side by side. Table 3 summarizes the number of facilities selected, new CHCs opened, closures, coverage, and travel metrics across Models M1 (maximize coverage), M2 (minimize distance), and M3 (balanced). This comparison highlights the trade-offs between equity and efficiency: while M1 achieves nearly universal coverage, it does so at the cost of longer travel times; M2 minimizes travel distance but leaves peripheral populations underserved; and M3 provides a compromise solution that balances both objectives.

**Table 3:** Comparison of baseline model outcomes (M1: coverage, M2: distance, M3: balanced)

Model	Total facilities selected	New CHCs opened	Existing Clinics closed	% demand served	Median travel distance (km)	Median travel time (minutes)
M1 – Max coverage	223	44	9	~ 100%	Higher than M2/M3	> 30
M2 – Min distance	223	(same as M1)	(same as M1)	Lower in peripheral areas	2.71	13.8
M3 – Balanced (α = 0.5)	223	Subset of M1	Subset of M1	98.5%	2.71	31.2

### Extended facility options

When the model was rerun allowing all 279 candidate sites to be considered, the optimal solution selected 225 facilities—just two more than in the baseline configuration. Table 4 shows that the addition of these facilities slightly reduced median travel times, particularly when the weighting favored coverage (α = 0.25). However, the marginal benefits for α = 0.5 and α = 0.75 were minimal, suggesting that expanding beyond 223 facilities yields diminishing returns relative to the investment required.

**Table 4:** Likely demand for weighted preferences (Model 3) allowing for additional facilities

Weighting	Optimal number of facilities	Median distance traveled (km)	Median time traveled (mins)	Maximum distance traveled (km)	Maximum time traveled (mins)	Interquartile range (km)
α = 0.25	225	2.64	13.4	21.55	109.6	1.21
α = 0.50	225	2.57	13.1	20.39	103.7	1.11
α = 0.75	225	2.57	13.1	20.39	103.7	1.11

### Varying demand scenarios

**Fig. 6:**
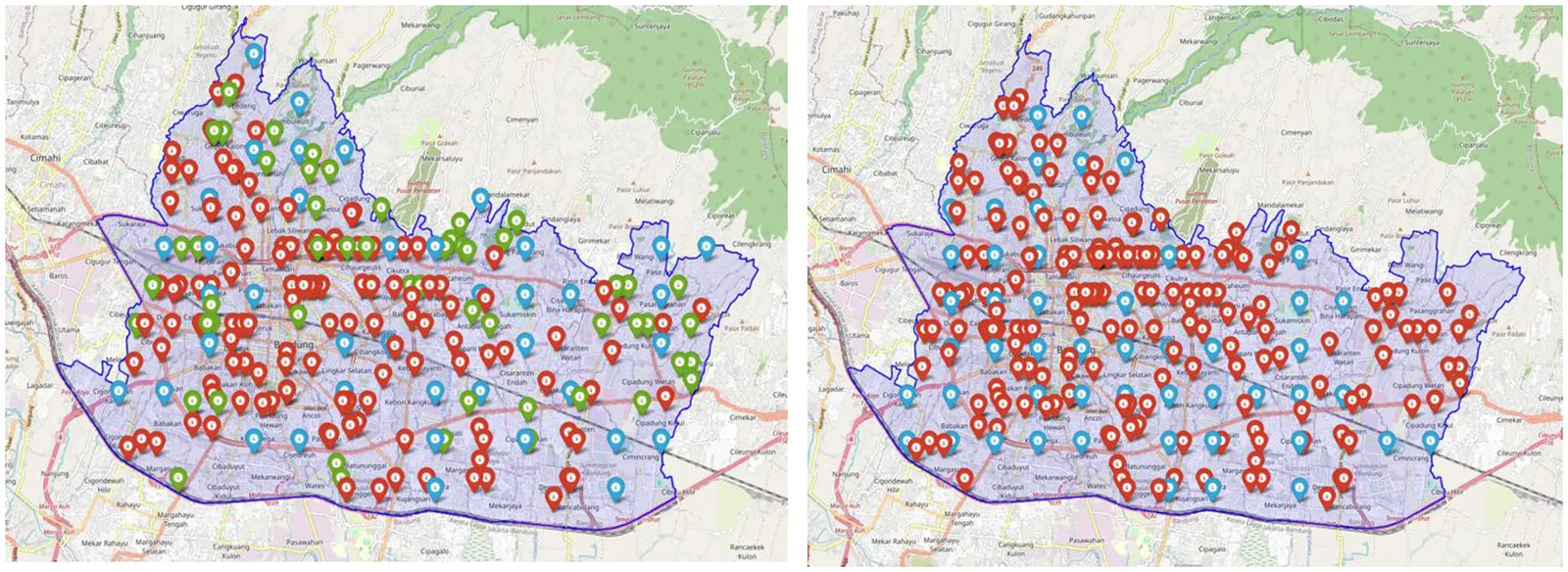
Facility allocations under low- (left) and high-demand (right) scenarios

We also tested the robustness of Model (M3) under alternative population forecasts. Under the low-demand scenario (− 33.4% relative to baseline), the model recommended only 168 facilities, including 38 new CHCs, while closing 58 existing facilities (primarily Clinics). This indicates that the current facility distribution is suboptimal even for present-day demand levels. Conversely, under the high-demand scenario (+ 10.5% relative to baseline), the model required 238 facilities, including 50 new CHCs, to maintain service standards. Figure 6 illustrates these contrasting facility allocations, with notable changes in peripheral areas and the composition of CHCs versus Clinics.

### Interpretation of findings

Taken together, the models highlight a clear trade-off between efficiency and equity. The minimum distance model (M2) provided highly efficient access for central populations but failed to adequately cover peripheral areas. The maximum coverage model (M1), while equitable, led to longer average travel times and a higher number of facilities. The balanced approach (M3) demonstrated that a compromise solution—covering 98.5% of the population with a median travel time of ~ 31 min—is achievable. Importantly, small expansions in facility numbers (as shown in Table 4) yielded limited additional benefit, suggesting that resource allocation must be carefully weighed against marginal gains.

### Comparison with previous literature

These findings are consistent with prior studies that have shown healthcare facility location problems often face trade-offs between equity and efficiency and exhibit diminishing returns once a critical threshold of facilities is reached [23, 32]. Unlike many earlier works that assume static demand, our approach explicitly integrates long-term population forecasts, providing a forward-looking perspective more relevant to strategic planning. Forecasting-driven facility location models have been applied in other sectors such as education [43] and emergency planning [44], but their application to primary healthcare planning in low- and middle-income countries remains limited. This study thus contributes to filling that gap by demonstrating how forecasting and optimisation can be integrated in data-limited but demographically dynamic contexts.

### Policy implications

For policymakers in West Java, the results suggest that investing in approximately 223 CHCs would be sufficient to balance equity and efficiency by 2045, while additional facilities offer limited incremental benefits. The results also underscore the importance of proactive planning: failing to anticipate population growth could leave peripheral communities underserved, whereas excessive expansion risks straining limited budgets for minimal gain. Incorporating these insights into regional planning can support progress toward Indonesia’s Universal Health Coverage goals.

### Limitations and future work

This study has several limitations. Financial costs—such as land acquisition, staffing, and equipment—were not included, and therefore investment trade-offs could not be fully evaluated. The models assumed uniform facility capacity and static utilisation rates, which simplify the complexity of real-world service provision. Moreover, demand was modeled deterministically, excluding stochastic fluctuations such as seasonal variation. Future work should address these limitations by incorporating cost functions, stochastic demand, and dynamic facility capacities. Additional stakeholder engagement and validation with empirical utilisation data would also strengthen the applicability of the framework.

### Conclusions, policy recommendations, and further work

This study aimed to address spatial inequities in access to primary healthcare services by integrating population forecasting with location-allocation modelling. Using Kota Bandung as a case study, we projected population growth to 2045 and estimated future daily demand for community health centres (CHCs), which was then matched with strategic facility placement across a high-resolution demand grid. The modelling produced several feasible CHC configuration scenarios that satisfy service coverage and access constraints, offering tangible insights for regional planning.

Theoretically, this work contributes to the limited literature on forward-looking healthcare facility location models by incorporating long-term population projections into optimisation-based planning. This integration advances the methodological understanding of how spatial demand forecasting can be linked with facility siting to guide decisions under demographic uncertainty. The study also highlights the utility of demand grids as a practical abstraction when granular utilisation data are unavailable.

Practically, the modelling framework offers a flexible and scalable tool to support strategic planning efforts by regional governments. The results are currently being used to inform discussions with the West Java Provincial Health Office as they prepare for substantial primary care infrastructure investment. By identifying robust CHC location plans under multiple policy constraints, this study provides decision-makers with evidence-based guidance that can be readily adapted to evolving priorities or geographic contexts.

However, several limitations must be acknowledged. First, the lack of real-time CHC utilisation data constrained demand estimation and validation. Second, financial cost components were omitted, which limits the analysis of investment trade-offs. Third, simplifying assumptions—such as equal facility capacities, fixed utilisation rates, and static travel speeds—were adopted to make the problem tractable. Future work could address these limitations by integrating cost models, collecting utilisation data at finer spatial scales, and exploring stochastic or dynamic extensions to the modelling framework.

## Supplementary Information

Below is the link to the electronic supplementary material.

Supplementary Material 1

## Data Availability

The datasets used and/or analysed during the current study are derived from official administrative and publicly available sources as described in the article. Processed datasets and model outputs generated during the study have been deposited in the Cardiff University Research Data Repository 10.17035/cardiff.32608539.

## Abbreviations

ACF: Autocorrelation Function
ADF: Augmented Dicky‒Fuller
AHP: Analytical Hierarchical Process
AIC: Akaike Information Criterion
BIC: Bayesian Information Criterion
BPJS: Badan Penyelenggara Jaminan Sosial
CFLP: Capacitated Facility Location Problem
CHC: Community Healthcare Centres
CMCLP: Capacitated Maximal Covering Location Problem
HQIC: Hannan–Quinn Information Criterion
MCLP: Maximal Coverage Location Problem
MILP: Mixed-Integer Linear Programming
PACF: Partial Autocorrelation Function
UFLP: Uncapacitated Facility Location Problem
