# Supplementary material for: Reducing healthcare access inequities in West Java through forecasting and location-allocation models
Source: BMC Health Serv Res. 2026 Jun 10;26:1146. doi: 10.1186/s12913-026-14889-1 (PMC13488113; doi:10.1186/s12913-026-14889-1)
Supplement: Supplementary file 1 [file 12913_2026_14889_MOESM1_ESM.docx]

**Appendix 1**

Figure A1: First-Order Differenced Log-Transformed Population of Kota Bandung 1950--2023

Figure A2: Second-Order Differenced Log-Transformed Population of Kota Bandung 1950--2023

Figure A3b: Partial-Autocorrelation Function of the Transformed Time Series

Figure A3a: Autocorrelation Function of the
Transformed Time Series
